# Effects of Nasal Continuous Positive Airway Pressure on Cerebral Hemodynamics in Preterm Infants

**DOI:** 10.3389/fped.2020.00487

**Published:** 2020-08-21

**Authors:** Han Zhou, Xuewen Hou, Rui Cheng, Youyan Zhao, Jie Qiu

**Affiliations:** ^1^Department of Newborn Infants, Children's Hospital of Nanjing Medical University, Nanjing, China; ^2^Department of Paediatrics, Nantong First People's Hospital, Nantong, China

**Keywords:** cerebral hemodynamics, nasal continuous positive airway pressure, near-infrared spectroscopy, pressure, preterm infant

## Abstract

**Background:** To evaluate the effects of pressure levels on cerebral hemodynamics in premature infants receiving nasal continuous positive airway pressure (nCPAP) during the first 3 days of life.

**Methods:** Forty-four preterm infants treated with nCPAP were divided into two groups: very preterm infants [gestational age 1 (GA1), GA < 32 weeks, *n* = 24] and moderate/late preterm infants (GA2 group, GA 32–37 weeks, *n* = 20). During monitoring, pressure levels were set at 4 → 6 → 8 → 4 cmH_2_O, and cerebral hemodynamics was assessed by near-infrared spectroscopy (NIRS). Vital signs, peripheral oxygen saturation (SpO_2_) and transcutaneous carbon dioxide pressure (TcPCO_2_) were simultaneously recorded.

**Results:** Pressures of 4–8 cmH_2_O had no significant influence on cerebral hemodynamics, TcPCO_2_, SpO_2_ or other vital signs. The tissue oxygenation index (TOI), the difference between oxygenated hemoglobin (ΔHbO_2_) and deoxygenated hemoglobin (ΔHHb) (ΔHbD), and cerebral blood volume (ΔCBV) were all significantly positively correlated with gestational and post-natal age, with fluctuations being greater in the GA1 group. ΔHbD and ΔCBV were also significantly positively correlated with TcPCO_2_.

**Conclusions:** No significant differences were observed in cerebral hemodynamics when the nCPAP pressure was set to 4–8 cmH_2_O.

## Introduction

Neonatal respiratory distress syndrome (RDS) is a common respiratory disorder in premature infants. Non-invasive respiratory support is the optimal respiratory support method for RDS. Nasal continuous positive airway pressure (nCPAP), one of the most common non-invasive assisted ventilation methods, is widely used in the neonatal intensive care unit (NICU). Although management of RDS is improving, nCPAP is still a crucial therapeutic measure of RDS (1).

In recent years, increasing numbers of researchers are paying attention to the effects of nCPAP on cerebral hemodynamics (2–4). Some researchers consider the pressure of nCPAP to increase intrathoracic pressure, impede systemic and pulmonary venous return (5), and decrease cardiac output (CO) (6), which may ultimately decrease cerebral perfusion. Egwu et al. demonstrated that nCPAP might represent an independent predictor for intraventricular hemorrhage (IVH) in early preterm neonates (7). There is currently disagreement on the optimal nCPAP pressure for preterm

RDS, and 4–6 cmH_2_O is a common range applied in the NICU (3, 4). However, some researchers prefer to use 8 cmH_2_O or even higher pressures (8) because 8 cmH_2_O has larger end-expiratory volume and tidal volume, as well as a lower respiratory rate and thoracoabdominal asynchrony than when using 2–6 cmH_2_O (8). Therefore, in premature infants, it is important that clinically utilized nCPAP pressures are safe for the developing preterm brain. The cost-benefit ratio needs to be weighed to choose an appropriate nCPAP pressure.

Although it has been demonstrated that the use of nCPAP decreases cerebral perfusion in healthy adult volunteers (9), study results in preterm infants are distinct. Dani et al. found that nCPAP pressures of 2–6 cmH_2_O did not affect cerebral oxygenation or cerebral blood volume in RDS preterm infants with <30 weeks' gestation during the first 10 days of life (3). Bembich S et al. also found no obvious fluctuations in CBF in preterm newborns of 26–33 weeks' gestation whose ages at observation were 2–21 days (median age was 6 days) using nCPAP with pressures of 3–8 cmH_2_O (4). It is well-known that the first 3 days after birth are important for RDS treatment, and application of nCPAP during this period is vital to cure premature infants with RDS. However, during this period, the CBF of premature infants is very unstable, which may cause IVH, hypoxic-ischemic brain damage and other brain injuries. It is important to understand the adaptive changes in the cerebral circulation of premature infants during the first 3 days of life (10, 11). Therefore, in this study, we investigated the effects of nCPAP pressures on cerebral hemodynamics in premature infants during the first 3 days of life.

Preterm infants receiving nCPAP within the first 3 days of life were selected for examination of cerebral hemodynamics using near-infrared spectroscopy (NIRS) when nCPAP pressures varied from 4 to 8 cmH_2_O. We focused on assessing the safety and stability of nCPAP on cerebral hemodynamics in premature infants during the first 3 days of life. The purpose of this study was to provide a theoretical basis for selecting safe and effective nCPAP pressure levels in premature infants with RDS soon after birth.

## Materials and Methods

### Participants

This study was conducted in the NICU of Children's Hospital of Nanjing Medical University from April 2018 to October 2018. Inclusion criteria were as follows: (1) preterm infants with gestational age (GA) <37 weeks; (2) preterm infants diagnosed with RDS and receiving nCPAP within 3 days after birth; and (3) Apgar scores > 7 at 1, 5, and 10 min. Exclusion criteria were one or any combination of the following: (1) peripheral oxygen saturation (SpO_2_) not maintained in the normal range during the monitoring process; (2) infants did not stay calm during the monitoring process; (3) serious brain injuries caused by asphyxia, birth injury, intrauterine infection or others; (4) genetic metabolic diseases; and (5) serious congenital heart disease, nervous system malformation or other congenital diseases or serious complications. This study was approved by the ethics committee of Children's Hospital of Nanjing Medical University, and consent was obtained from all infants' parents.

All enrolled infants were divided into 2 groups according to GA. The first group comprised very preterm infants (GA1 group, GA < 32 weeks, *n* = 24) with the following distributions: 1-day group: monitoring within 24 h after birth (*n* = 7); 2-days group: monitoring between 24 and 48 h after birth (*n* = 9); and 3-days group: monitoring between 48 and 72 h after birth (*n* = 8). The second group comprised moderate to late preterm infants (GA2 group, GA 32–36^+6^ weeks, *n* = 20) with the following distributions: 1-day group: monitoring within 24 h after birth (*n* = 6); 2-days group: monitoring between 24 and 48 h after birth (*n* = 7); 3-days group: monitoring between 48 and 72 h after birth (*n* = 7).

### Procedures

NCPAP was provided by the Stephan CPAP system (Stephan; Rheinland-Pfalz, Gackenbach, Germany). The pressure was initially set at 4 cmH_2_O, and data were recorded for 30 min. Subsequently, the pressure was raised to 6 and 8 cmH_2_O for an additional 30 min each of recording. Last, the pressure was returned to 4 cmH_2_O for another 30 min. The nCPAP pressure was kept constant during each time point. All infants were in the supine position with mouth closed using pacifiers and were quiet or sleeping during the monitoring process.

### Outcomes

The CBF and oxygenation, the primary outcomes, were continuously monitored from the beginning to the end of the procedures using NIRS (EGOS-600A, EnginMed; Suzhou, Jiangsu, China). During the recording time, the tissue oxygenation index (TOI), deoxygenated hemoglobin (ΔHHb), oxygenated hemoglobin (ΔHbO_2_) and total hemoglobin (ΔtHb) were recorded. Cerebral blood volume (ΔCBV) and the difference between ΔHbO_2_ and ΔHHb (ΔHbD) were calculated according to the following reported formula: ΔCBV = ΔtHb × 0.89/Hb and ΔHbD = ΔHbO_2_ – ΔHHb (3). Arterial hemoglobin (Hb) was measured within 12 h before beginning the study.

Secondary outcomes were transcutaneous carbon dioxide tension (TcPCO_2_), SpO_2_, and other vital signs. TcPCO_2_ was measured using a TCM4 Combim (Radiometer; Brea, California, United States). For non-invasive monitoring of SpO_2_, heart rate (HR) and respiratory rate (RR), a pulse oximeter probe (N-300®, Nellcor; Minneapolis, Minnesota, United States) was placed around the right wrist/hand. The mean systemic arterial blood pressure (MABP) was measured non-invasively every 30 min using a neonatal cuff around the slightly outstretched left upper arm. Vital signs (SpO_2_, HR, RR and MABP) were recorded every 30 min by using an IntelliVue MP50 Monitor (Philips; Amsterdam, Netherlands).

### Statistical Analysis

No a priori sample size was determined. However, this sample of 44 preterm infants gives ~75% power to detect at least a 10% difference in CBF. SPSS 22.0 was used for statistical analysis. Normally distributed data are presented as the mean value ± standard deviation ($\bar{x}$ ± *s*), and the *t*-test was used for comparison between two groups. Analysis of variance was used to compare multiple groups, and if the difference was statistically significant, the *post-hoc* Bonferroni test was used for further comparison. Continuous data are presented as the number of cases or percentages (%), and Fisher's exact test was used for comparison between two groups. Pearson correlation was conducted to analyze correlations in the data, and *P* < 0.05 was considered statistically significant.

## Results

### Demographic Variables

**Figure 1:**
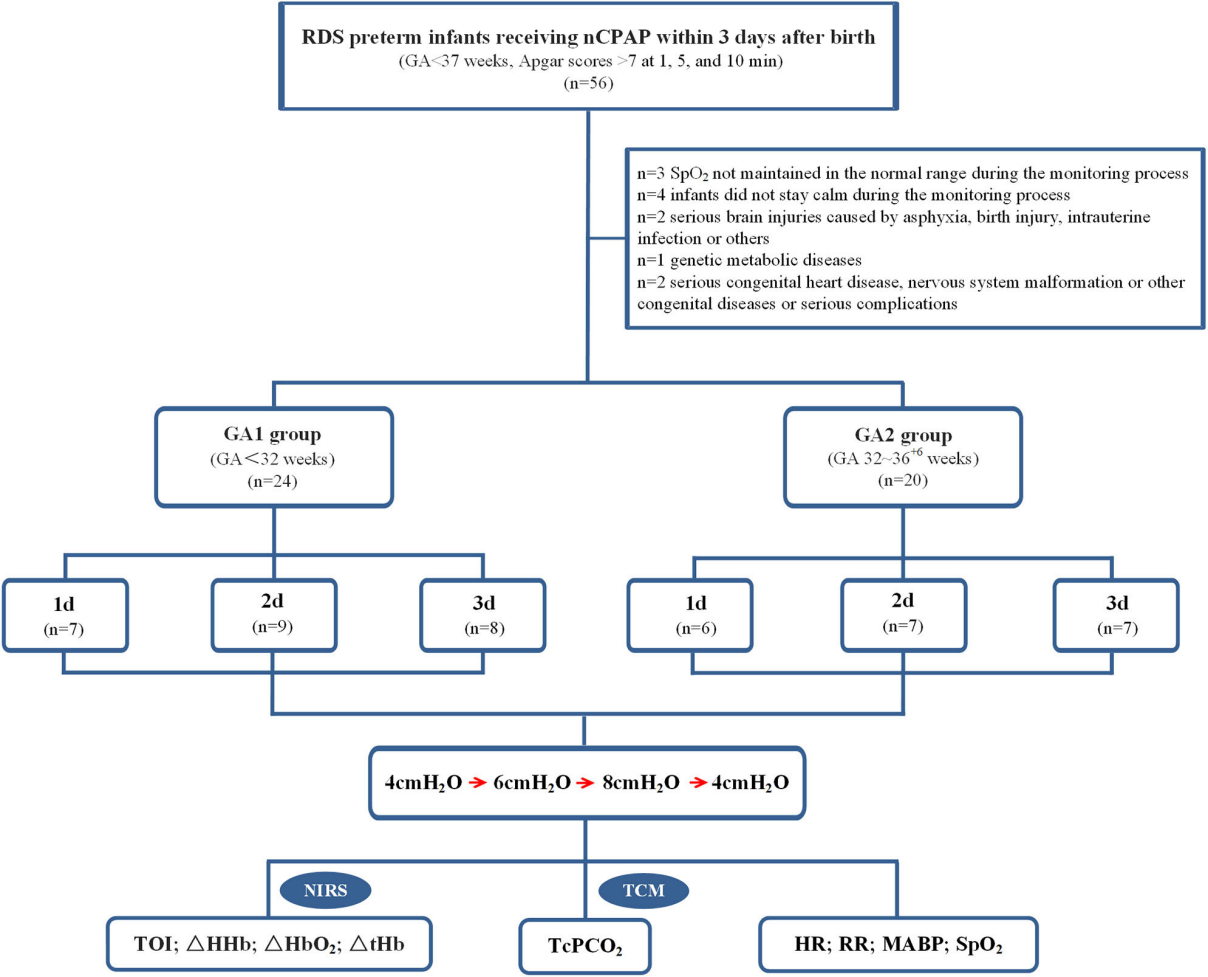
Study profile and patient flow.

From April 2018 to October 2018, 44 preterm infants receiving nCPAP were enrolled in this study (flow diagram, Figure 1). Clinical and demographic data are presented in Table 1. Except for GA and birth weight (*P* = 0.00), there were no significant differences in sex, multiple births, mode of delivery, Apgar scores, prenatal use of glucocorticoids, premature rupture of fetal membranes or initial HR, RR, and MABP between the two groups (*P* > 0.05). Among the subgroups in each group, there were no significant differences in any variables (*P* > 0.05).

**Table 1:** Clinical and demographic characteristics of neonates.

	**Very preterm infant group (*****n*** **=** **24)**			**Moderate and late preterm group (*****n*** **=** **20)**		
	**1 day group (*n* = 7)**	**2 days group (*n* = 9)**	**3 days group (*n* = 8)**	**1 day group (n = 6)**	**2 days group (*n* = 7)**	**3 days group (*n* = 7)**
Sex (Male/Female)	3/4	5/4	4/4	3/3	4/3	3/4
Multiple births [*n* (%)]	1 (14.3)	3 (33.3)	2 (25.0)	1 (16.7)	1 (14.3)	2 (28.6)
Gestational age ($\bar{x}$ ±*s*, weeks)	28.6 ± 1.8	29.0 ± 1.5	28.8 ± 1.5	34.4 ± 0.8*	33.6 ± 1.5*	33.3 ± 3.6*
Birth weight ($\bar{x}$ ±*s*, g)	1174 ± 170	1314 ± 281	1161 ± 209	2151 ± 125*	2070 ± 184*	1913 ± 298*
Cesarean [*n* (%)]	3 (42.9)	6 (66.7)	6 (75.0)	4 (66.7)	5 (71.4)	5 (71.4)
1 min Apgar ($\bar{x}$ ±*s*)	8.0 ± 1.0	7.7 ± 1.8	7.6 ± 2.1	8.3 ± 1.0	8.7 ± 1.1	8.6 ± 0.8
5 min Apgar ($\bar{x}$ ±*s*)	8.9 ± 0.7	7.7 ± 0.6	8.8 ± 0.5	9.3 ± 1.0	9.4 ± 0.5	9.3 ± 0.5
Prenatal use of glucocorticoids [*n* (%)]	4 (57.1)	6 (66.7)	5 (62.5)	3 (50.0)	4 (57.1)	3 (42.9)
Premature rupture of fetal membranes >24 h [*n* (%)]	4 (57.1)	4 (44.4)	3 (37.5)	2 (33.3)	5 (71.4)	3 (42.9)
HR ($\bar{x}$ ± s)	145 ± 7	144 ± 8	142 ± 6	142 ± 8	143 ± 10	140 ± 7
RR ($\bar{x}$ ± s)	46 ± 8	45 ± 6	43 ± 7	43 ± 5	45 ± 8	42 ± 6
MABP ($\bar{x}$ ± s)	38 ± 8	40 ± 7	42 ± 5	42 ± 6	43 ± 7	45 ± 5

Gestational age and birth weight of GA2 group were all significant higher than that of GA1 group at the same post-natal age (*P < 0.05).

### Effects of Different nCPAP Pressure Levels on Cerebral Hemodynamics, TcPCO_2_, SpO_2_, and Other Vital Signs

**Figure 2:**
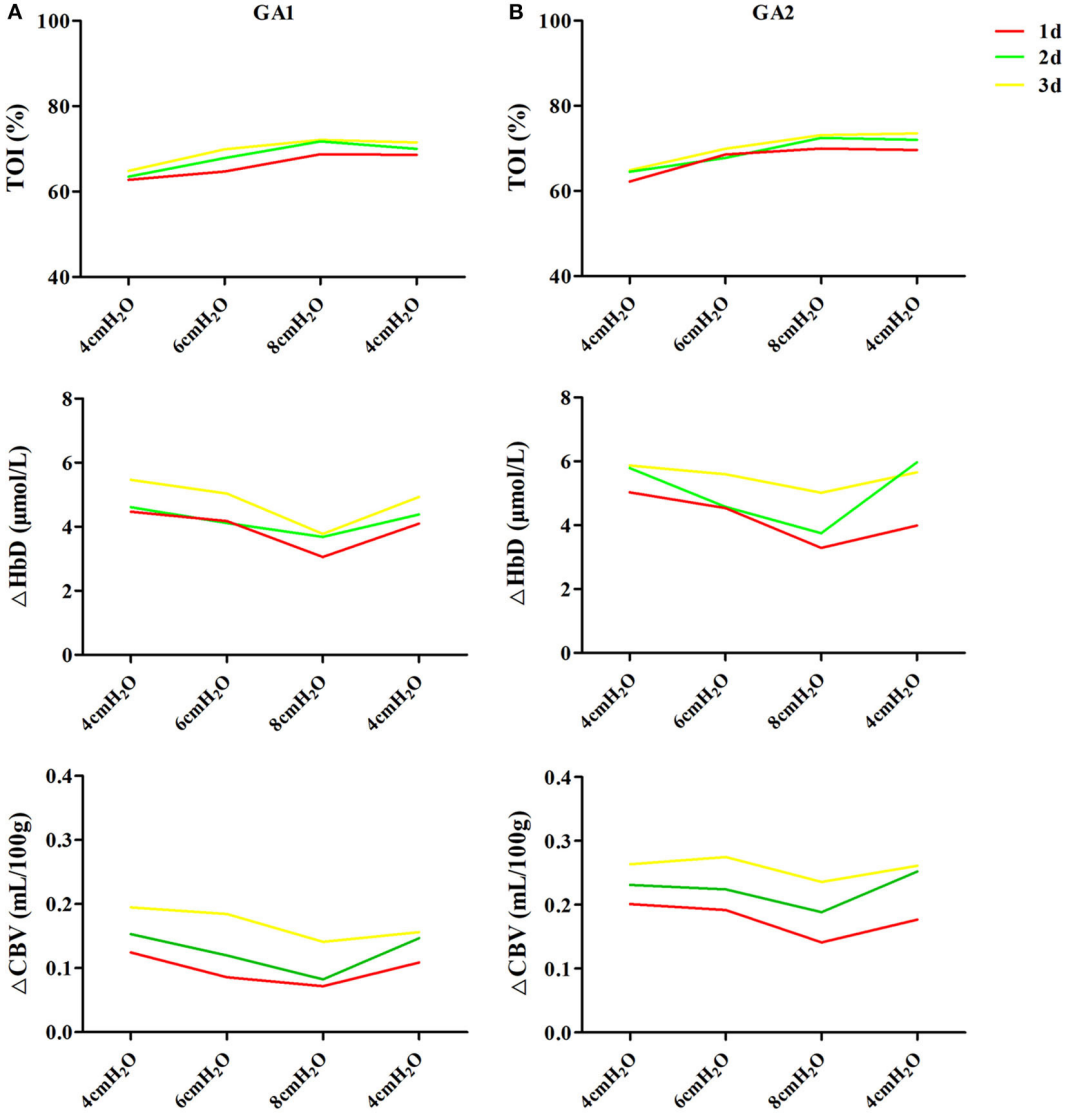
Effects of nCPAP pressure levels on cerebral hemodynamics. NCPAP pressures of 4–8 cmH_2_O had no significant influence on TOI, ΔHbD or ΔCBV in either very preterm infants **(A)** or moderate to late preterm infants **(B)** (*P* > 0.05).

**Figure 3:**
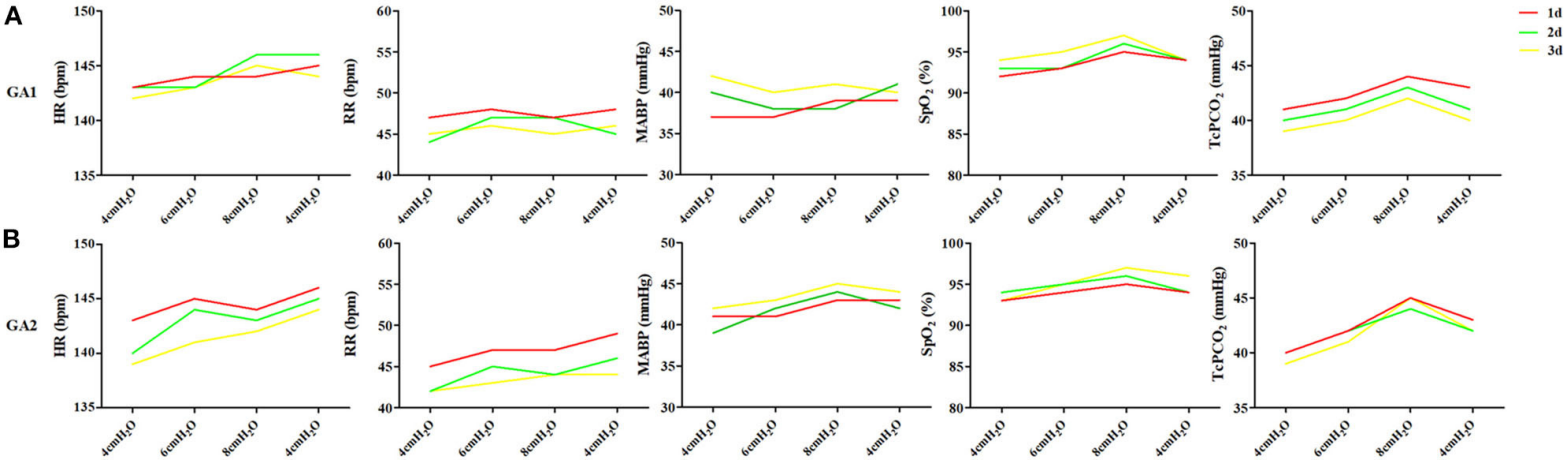
Effects of nCPAP pressure levels on TcPCO_2_, SpO_2_ and other vital signs. NCPAP pressures of 4–8 cmH_2_O had no significant influence on TcPCO_2_, SpO_2_ or other vital signs in either very preterm infants **(A)** or moderate to late preterm infants **(B)** (*P* > 0.05).

In enrolled infants, the baseline set nCPAP pressure was 4 cmH_2_O, and FiO_2_ was 0.21–0.25 to maintain SpO_2_ at 92–96%. The pressure, which was set from 4 to 8 cmH_2_O, had no significant influence on cerebral hemodynamics either in very preterm infants (Figure 2A) or in moderate to late preterm infants (Figure 2B). Though ΔHbD and ΔCBV tended to decline at 8 cmH_2_O, the difference was not significant (*P* > 0.05). Furthermore, in the very preterm infant (Figure 3A) and the moderate to late preterm infant (Figure 3B) groups, nCPAP pressure levels of 4–8 cmH_2_O had no significant influence on TcPCO_2_, SpO_2_ or other vital signs (*P* > 0.05).

### Related Factor Analysis of Cerebral Hemodynamics

**Figure 4:**
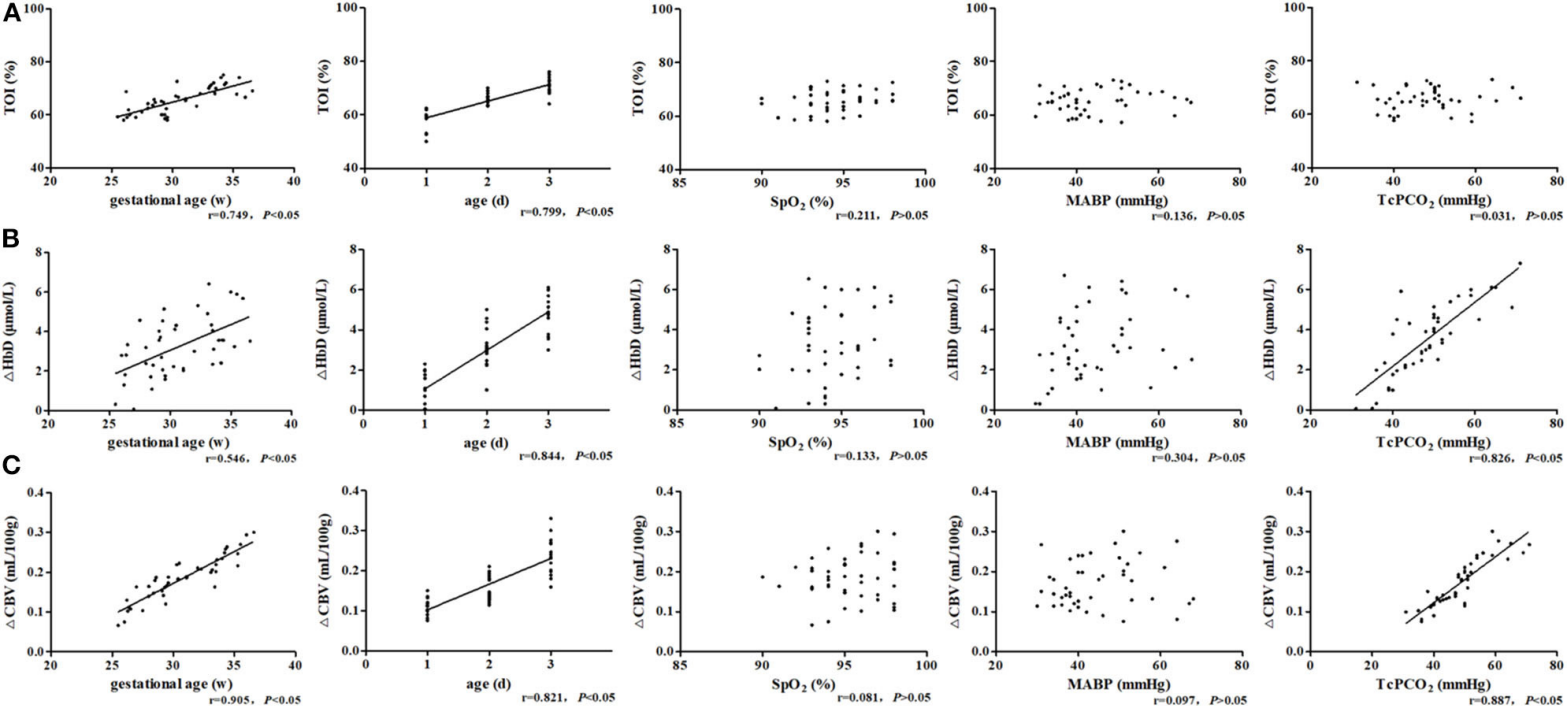
Related factor analysis of cerebral hemodynamics. **(A)** TOI was significantly positively correlated with GA (*r* = 0.749, *P* < 0.05) and post-natal age (*r* = 0.799, *P* < 0.05). **(B)** ΔHbD was significantly positively correlated with GA (*r* = 0.546, *P* < 0.05), post-natal age (*r* = 0.844, *P* < 0.05), and TcPCO_2_ (*r* = 0.826, *P* < 0.05). **(C)** ΔCBV was significantly positively correlated with GA (*r* = 0.905, *P* < 0.05), post-natal age (*r* = 0.821, *P* < 0.05), and TcPCO_2_ (*r* = 0.887, *P* < 0.05).

The TOI was significantly positively correlated with GA (*r* = 0.749, *P* < 0.05) and post-natal age (*r* = 0.799, *P* < 0.05) (Figure 4A). ΔHbD was significantly positively correlated with GA (*r* = 0.546, *P* < 0.05), post-natal age (*r* = 0.844, *P* < 0.05), and TcPCO_2_ (*r* = 0.826, *P* < 0.05) (Figure 4B). Similarly, ΔCBV was significantly positively correlated with GA (*r* = 0.905, *P* < 0.05), post-natal age (*r* = 0.821, *P* < 0.05), and TcPCO_2_ (*r* = 0.887, *P* < 0.05) (Figure 4C).

### Effects of GA and Post-natal Age on Cerebral Hemodynamics

**Figure 5:**
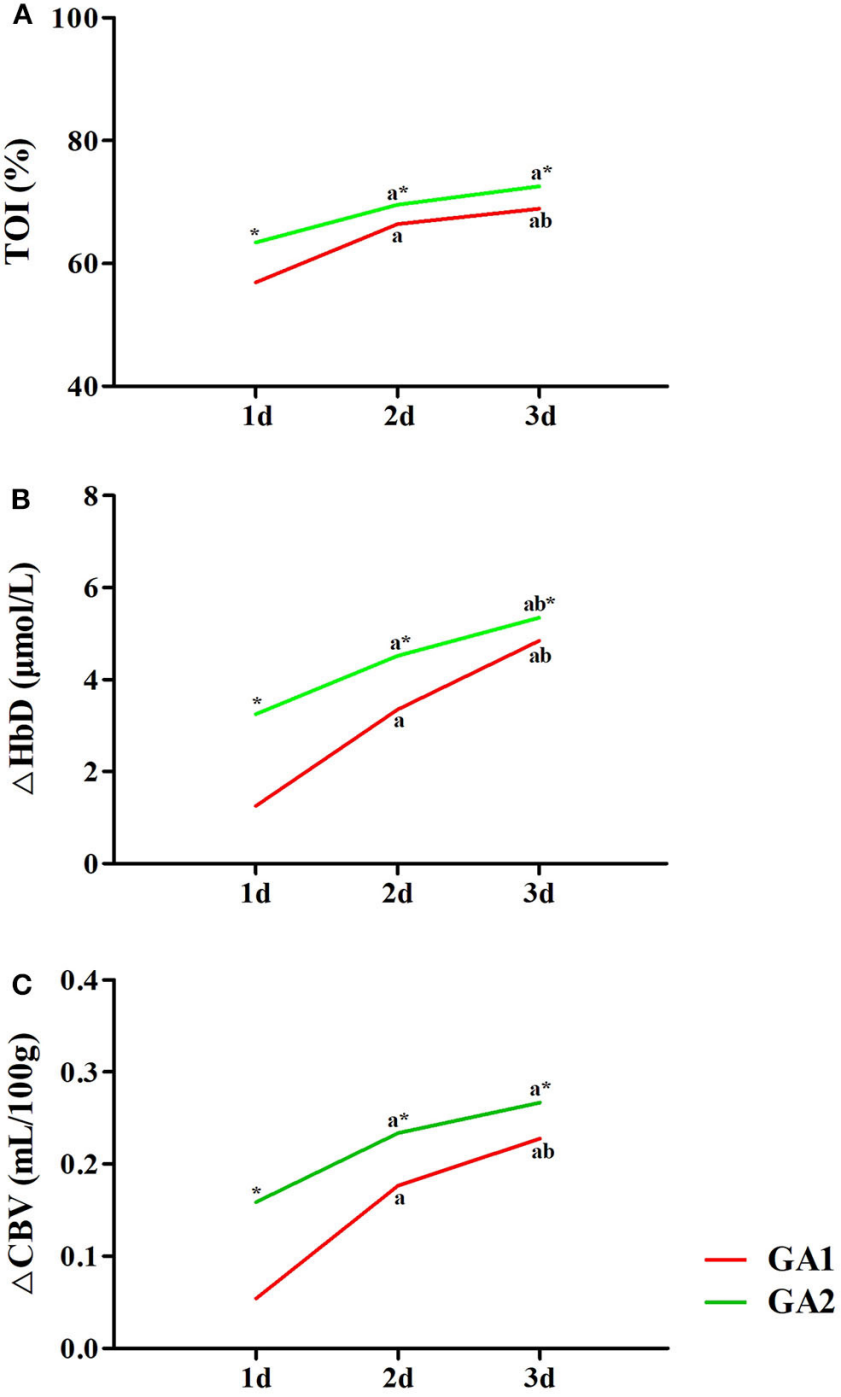
Effects of GA and post-natal age on cerebral hemodynamics. TOI **(A)**, ΔHbD **(B)**, and ΔCBV **(C)** in moderate to late preterm infants (GA2) were all significantly higher than in very preterm infants (GA1) at the same post-natal age (*P < 0.05). In both the GA1 and GA2 groups, TOI **(A)**, ΔHbD **(B)**, and ΔCBV **(C)** at 2 and 3 days were significantly higher than at 1 day (^a^*P* < 0.05). In the GA1 group, TOI **(A)**, ΔHbD **(B)**, and ΔCBV **(C)** at 3 days were all significantly higher than those at 2 days (^b^*P* < 0.05). In the GA2 group, only ΔHbD **(B)** at 3 days was significant higher than at 2 days (^b^*P* < 0.05).

As shown in Figure 5, the TOI, ΔHbD and ΔCBV in moderate to late preterm infants were all significantly higher than in very preterm infants at the same post-natal age (*P* < 0.05). The TOI, ΔHbD and ΔCBV of very preterm infants gradually increased during the first 3 days of life, and differences between the three groups were significant (*P* < 0.05). In moderate to late preterm infants, compared with the 1-day group, TOI and ΔCBV markedly increased in the 2- and 3-days groups (*P* < 0.05). However, TOI and ΔCBV were not significantly different between the 2- and 3-days groups (*P* > 0.05), while ΔHbD among the three groups exhibited significant differences (*P* < 0.05).

## Discussion

In this study, we evaluated the effects of nCPAP pressure on cerebral hemodynamics in premature infants, demonstrating several novel and interesting findings. First, nCPAP pressures of 4–8 cmH_2_O had no significant influence on the cerebral hemodynamics, vital signs, SpO_2_, or TcPCO_2_ of preterm infants. Second, TOI, ΔHbD, and ΔCBV were all significantly positively correlated with GA and post-natal age. ΔHbD and ΔCBV were also significantly positively correlated with TcPCO_2_. Finally, TOI, ΔHbD, and ΔCBV in moderate to late preterm infants were significantly higher than in very preterm infants at different post-natal ages. TOI, ΔHbD, and ΔCBV in the two groups all gradually increased during the first 3 days of life, and fluctuations in very preterm infants were even greater.

NCPAP, the most common respiratory support used in the NICU, delivers continuous positive air pressure into the airway to improve oxygenation by maintaining lung volume, increasing pulmonary functional residual capacity and reducing airway resistance. Some researchers think that nCPAP might decrease cerebral perfusion by increasing intrathoracic pressure, lowering CO, and increasing central venous and intracranial pressure (12–14). However, Beker et al. (15) found that the CO and MABP were unchanged at pressure levels of 4, 6, and 8 cmH_2_O in preterm infants. Moritz et al. (16) also demonstrated that nCPAP with a mean level of 4 cmH_2_O (up to 7 cmH_2_O) did not influence CO in preterm infants. Our results also supported that the nCPAP pressure that was set from 4 to 8 cmH_2_O did not significantly influence cerebral hemodynamics. We speculate that infants may be able to quickly compensate for increased intrathoracic pressure caused by pressure due to their highly compliant chest wall, which ultimately reduces the effect of pressure on cerebral hemodynamics (10, 11). Two studies found that nCPAP at pressure levels of 10 and 12 cmH_2_O decreased CBF in adults (9, 17). Hsu et al. (6) also observed that nCPAP affects neonatal CO when the nCPAP pressure was 10 cmH_2_O. These findings suggest that extra high pressure affects cerebral hemodynamics. However, our results indicated that pressure at a level of 8 cmH_2_O had no significant effect on cerebral hemodynamics in preterm infants. Hence, cerebral hemodynamics in premature infants remains relatively stable when the pressure is set within the range of 4–8 cmH_2_O.

The TOI is a parameter measured directly by NIRS and simply reflects the oxygen content in local brain tissue. ΔHbD is reported to be a powerful target that sensitively reflects CBF (18, 19). ΔCBV reflects cerebral blood volume and is correlated with CBF. Our results demonstrated that TOI, ΔHbD, and ΔCBV are all significantly positively correlated with GA and post-natal age, which is consistent with other reports (18, 20). TOI, ΔHbD, and ΔCBV in moderate to late preterm infants were significantly higher than in very preterm infants at different post-natal ages. Using Doppler color ultrasonography, Pezzati et al. found that with increased GA, the resistance index (RI) decreased, and cerebral blood flow velocity (CBFV) subsequently significantly increased in both the anterior cerebral artery and the right and left middle cerebral arteries (21). Therefore, we believe that cerebral oxygenation and CBF improve with increased GA through decreasing the RI of cerebral vessels and increasing CBFV. Consistent with previous findings (22), we also demonstrated that cerebral oxygenation and CBF are significantly correlated with post-natal age in preterm infants during the early days of life. The reasons for this may include increased CO, arterial ductal closure and decreased intracranial pressure after birth (23). Of note, although cerebral hemodynamics in both groups gradually increased during the first 3 days of life, fluctuations in very preterm infants were greater than those in moderate to late preterm infants. We speculate that premature infants of lower GA have weaker autoregulation of cerebral hemodynamics, leading to larger fluctuations in blood flow and a greater possibility of brain injury. Hence, clinicians need to avoid blood flow fluctuations in premature infants, especially those who are extremely preterm, during the first days of life to reduce the risk of brain injuries.

TcPCO_2_ is another related factor of cerebral hemodynamics. Our results monitored by TCM showed that TcPCO_2_ correlates with CBF indexes, including ΔHbD and ΔCBV. In recent years, more researchers have begun to realize the close relationship between carbon dioxide pressure (PaCO_2_) and brain injury in preterm infants. It has been demonstrated that either overly high or overly low PaCO_2_ can both damage cerebral autoregulation and increase fluctuations in CBF, causing brain injuries, such as IVH (24, 25). Greisen et al. reported significant neurological abnormalities at 18 months in very-low-birth-weight infants who endured severe hypocapnia within 24 h of life (26). In addition, Dix et al. (24) showed that acute increases in PaCO_2_ diminished brain activity, potentially leading to adverse neurological outcomes in preterm infants. Therefore, clinicians should also keep PaCO_2_ levels within a reasonable range and avoid its fluctuation during the first days of life to reduce the risk of brain injuries. Dix et al. (24) also found that acute increases in PaCO_2_ are associated with increased cerebral oxygenation. However, Naulaers et al. (20) found that the TOI was independent of PaCO_2_, consistent with our results. The exact relationship between PaCO_2_ and cerebral oxygenation requires further studies.

SpO_2_ is one of the most common and intuitive indicators used in the NICU. Some studies have found that higher SpO_2_ prevents brain damage through decreasing the incidence of intermittent apnea, hypoxemia and bradycardia (27, 28). However, our results did not reveal a correlation between SpO_2_ and cerebral hemodynamics. There are several potential reasons for this. There is a reported correlation between SpO_2_ and cerebral oxygenation when SpO_2_ is severely low (29). However, the values of SpO_2_ were high (91–98%) in our study, so this relationship between SpO_2_ and cerebral oxygenation was not apparent. In addition, high peripheral oxygenation does not necessarily lead to high cerebral oxygenation in preterm infants (4). Thus, SpO_2_ may not represent cerebral oxygenation and CBF precisely in preterm infants.

In this study, MABP did not obviously affect cerebral oxygenation or CBF. MABP is reported to change CBF when MABP is below 30 mmHg (30), but infants have an autoregulatory ability to maintain stable CBF when MABP is in a certain range, such as above 30 mmHg (31, 32). Therefore, some neonatologists contend that maintaining MABP above 30 mmHg may reduce the incidence of cerebral white matter lesions and IVH (33, 34). In addition, Michelet et al. found that MABP does not markedly influence cerebral oxygenation and CBF unless it has large fluctuations (35). In this study, MABPs in infants were all above 30 mmHg and did not exhibit large fluctuations, which may be the reason why no correlation between MABP and cerebral hemodynamics was observed.

One limitation of this study is that infants were not repeatedly measured but different infants were recruited for the different measurement time points. Because infants in this study were all pre-matures, in ethical terms, in order to avoid adverse effects of frequent operation on the same newborn especially very preterm infants for three consecutive days, we didn't measure the cerebral hemodynamics in the same patient. However, except GA and birth weight, there were no significant differences in the clinical and demographic data to rid the different infants' problem. Second, this is a pilot study with a small sample size intended to illustrate basic findings and we didn't do the sample size calculation in advance. In the near future, for more precise results, we will calculate the sample size and establish a blank control group for a larger study. In addition, besides NIRS, cerebral hemodynamic parameters will be observed using transcranial Doppler flow measurements in future research and long-term follow-up is needed.

In conclusion, nCPAP used at 4–8 cmH_2_O pressure levels did not affect cerebral hemodynamics. Furthermore, cerebral hemodynamics in preterm infants with lower GA or post-natal age was more unstable, and maintaining the stability of TcPCO_2_ may be crucial for reducing fluctuations in CBF.

## Data Availability Statement

The raw data supporting the conclusions of this article will be made available by the authors, without undue reservation.

## Ethics Statement

This study was approved by the ethics committee of Children's Hospital of Nanjing Medical University and achieved agreements from infants' parents. Written informed consent to participate in this study was provided by the participants' legal guardian/next of kin.

## Author Contributions

JQ and YZ conceptualized and designed the study, coordinated and supervised the experiments, provided the research materials/reagents, reviewed, and revised the manuscript. HZ conducted the experiments and collected data. XH analyzed the data and drafted the initial manuscript. RC was involved in data interpretation and manuscript preparation. All authors approved the final manuscript for submission and agreed to be accountable for all aspects of the work.

## Conflict of Interest

The authors declare that the research was conducted in the absence of any commercial or financial relationships that could be construed as a potential conflict of interest.

## Abbreviations

nCPAP: nasal continuous positive airway pressure
NICU: neonatal intensive care unit
GA: gestational age
RDS: respiratory distress syndrome
SpO_2_: peripheral oxygen saturation
NIRS: near-infrared spectroscopy
TOI: tissue oxygenation index
ΔHbO_2_: oxygenated hemoglobin
ΔHHb: deoxygenated hemoglobin
ΔtHb: total hemoglobin
ΔCBV: cerebral blood volume
ΔHbD: difference between ΔHbO_2_ and ΔHHb
Hb: hemoglobin
HR: heart rate
RR: respiratory rate
MABP: mean systemic arterial blood pressure
TcPCO_2_: transcutaneous carbon dioxide pressure
IVH: intraventricular hemorrhage
MV: mechanical ventilation
PS: pulmonary surfactant
EEG: electroencephalography
CBF: cerebral blood flow
CBFV: cerebral blood flow velocity
RI: resistance index
CO: cardiac output.
